# Knowledge, Attitude and Practice Regarding *Staphylococcus pettenkoferi*

**DOI:** 10.3390/idr14010015

**Published:** 2022-02-11

**Authors:** Marta Kierzkowska, Kinga Markowska, Anna Majewska

**Affiliations:** Department of Medical Microbiology, Medical University of Warsaw, Chalubinskiego 5 Str., 02-004 Warsaw, Poland; mkierzkowska@wum.edu.pl (M.K.); kmarkowska@wum.edu.pl (K.M.)

**Keywords:** bacteraemia, bloodstream infection, microbiological diagnostics, MALDI-TOF, *Staphylococcus pettenkoferi*

## Abstract

*Staphylococcus pettenkoferi* is a coagulase-negative staphylococcus, first described in 2002. Using medical databases, i.e., Scopus, Web of Science, Pubmed, and Embase, we identified and analysed research, reports, and opinions dealing with *S. pettenkoferi*. Published data allow us to conclude that *S. pettenkoferi* is a human commensal, opportunistic bacterium and may be isolated from the environment and animals. The involvement of *S. pettenkoferi* in bloodstream infection and osteomyelitis has been described, but its clinical relevance is not fully understood, so far. This work summarizes knowledge about *S. pettenkoferi* and reveals the difficulties and rules for interpreting the results of microbiological tests, when *S. pettenkoferi* has been identified in the blood sample. Clinical and laboratory criteria, recommended by Centers for Disease Control and Prevention (CDC) and the third international consensus definitions of sepsis and septic shock (Sepsis-3), are important to determine whether the presence of bacteria in the sample is a consequence of an infection, contamination from the environment, or translocation of the bacteria outside the place of its natural existence. The precise identification of bacteria from the blood sample and recognizing the true bacteraemia are critical to implement the appropriate procedures and make decisions concerning the patient’s medical care.

## 1. Introduction

*Staphylococcus pettenkoferi* is a coagulase negative staphylococcus (CoNS), whose clinical relevance is not fully understood. CoNS comprises of a heterogeneous group, ranging from non-pathogenic to pathogenic species. They differ in the virulence factors equipment. Most species are part of the healthy human skin and mucous membranes’ microbiota; a few have been regularly associated with human infections. Some species can contaminate specimens obtained from non-sterile and sterile sites of the human body [1,2]. It has been shown that CoNS are one of the major health care-associated pathogens. In certain circumstances, they are capable of causing opportunistic infections, mainly in patients with a peripheral or central catheter, urinary catheter, prosthetic heart valves (infective endocarditis), joint prostheses, and immunosuppressed patients [1,3,4,5]. CoNS have been assumed to be the leading agent of nosocomial bloodstream infections (BSIs), in particular, catheter-related bloodstream infection (CR-BSI) [1,5]. Additionally, they play a role in horizontal transfer of genes for antimicrobial resistance [1,6,7,8]. A separate issue is the difficulty in interpreting the microbiological test results, when CoNS were isolated from the site of infection, especially from the blood culture [9].

This study summarizes knowledge about *S. pettenkoferi* and, above all, evaluates the value of positive blood cultures for *S. pettenkoferi*.

Using medical databases (Scopus, Web of Science, Pubmed, and Embase), we identified 32 reports and opinions, published between 2002–2021, dealing with *S. pettenkoferi*. In seven reports, *S. pettenkoferi* in the sample of blood was related to infection by this bacteria, not to the contamination of the sample.

## 2. What Do We Actually Know about *Staphylococcus pettenkoferi*?

*S. pettenkoferi* is CoNS, first described in 2002 [3,10]. Since then, there have been several reports describing the involvement of *S. pettenkoferi* in blood infections and osteomyelitis, mainly in immunocompromised patients or patients with comorbidities, among which diabetes mellitus appears to be the most frequently observed [3,4,9,11,12,13,14,15,16,17]. *S. pettenkoferi* was also isolated from other sources, such as wounds and ulcers, vaginal abscesses, and frontal sinus—in all cases, with other organisms isolated in culture [17]. *S. pettenkoferi* was isolated from the animal environment (a feeding dish and blanket in a pet cage) [18], from buccal samples of two rabbits, a cat, and a dog [19], as well as from a cat with peritonitis [20] from airborne dust [7,13], and was found in the dust samples from the swine farms [8]. The first difficulty, regarding *S. pettenkoferi*, that researchers or staff of microbiological laboratories faced was the ability to identify this species accurately [10]. *S. pettenkoferi* is not very biochemically active; therefore, tests based on the analysis of biochemical features (i.e., ID 32 STAPH or Vitek 2 system) did not identify this species [10,14,21,22,23]. *S. pettenkoferi* phenotypically resembles *Staphylococcus capitis* subsp. *capitis*, *Staphylococcus auricularis* and *Staphylococcus warneri* [3,4,10,13,14]. This microorganism, based on biochemical tests (e.g., API and bioMérieux) has been incorrectly classified as *Kocuria* sp. or *Micrococcus* sp. [15]. Nowadays, microbiological laboratories are equipped with such devices and technologies as matrix-assisted laser desorption ionization time-of-flight (MALDI-TOF), enabling the identification of *S. pettenkoferi* from the clinical samples. Admittedly, the MALDI-TOF MS (Vitek MS) database v. 2.0 (bioMérieux, Marcy l’Etoile, France) did not allow an accurate identification of *S. pettenkoferi* [14]. Just an extension of the database to v. 3.0 enables precise identification of this species [4,22]. The bacteria may be also identified by MALDI BioTyper 2.3 and 3.0 (and newer versions of database) or Microflex LT RUO (both by Brucker Daltonics, Bremen, Germany) [11,14,15]. Thus, the mass spectrometry system is successfully adapted for routine identification in clinical microbiology laboratories [21,24]. The so-called golden standard in bacteria identification to the species level is 16S rRNA gene sequencing [14]. A relatively high cost of this method limits its application in routine microbiology diagnostics [25]. In recent times, more and more researchers are interested in the clinical significance of *S. pettenkoferi*. Cases of infection have been reported by researchers in various regions of the world [16,17,22,26]. Pathogenicity and virulence of this bacterium were also studied. So far, it has been shown that *S. pettenkoferi* can proliferate in phagocytes and murine macrophages. The ability to persist inside macrophages can be critical in systemic infections. Although *S. pettenkoferi* is relatively a slow growing *staphylococcus* (generation time is 74 min), it can produce at least the same amount of biofilm as *Staphylococcus* *aureus*. Biofilm-encoding genes (i.e., icaABCD and rsbUVW) and regulator-encoding genes (i.e., agr, mgrA, sarA, and seaS), also occurring in *S. aureus*, were identified by the whole-genome analysis of *S. pettenkoferi* strain. These features confirm a clinical relevance of this bacterium [16].

## 3. *S. pettenkoferi bacteraemia*—True Pathogen or Contamination?

**Figure 1:**
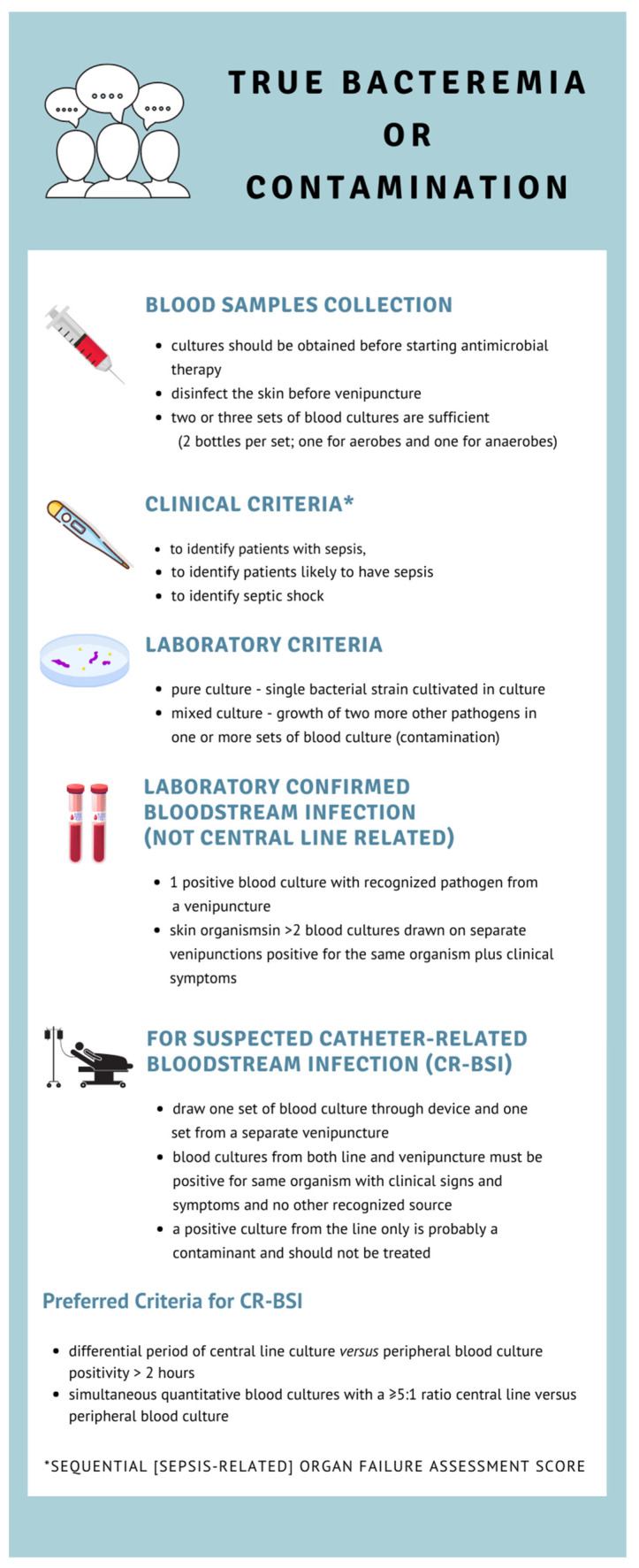
The Centers for Disease Control’s criteria for bloodstream infection (BSI) [27].

The answer to this question may be difficult, as it requires taking into account the clinical and laboratory criteria recommended by the Centers for Disease Control and Prevention (CDC) and third international consensus definitions of sepsis and septic shock (Figure 1) [27,28].

Based on the published information, it can be assumed that *S. pettenkoferi* inhabits human skin as a commensal [24]. In certain circumstances, it may, therefore, contaminate blood samples and other specimens [25]. Researchers have critically interpreted the cases of patients from whom *S. pettenkoferi* was isolated. They have tried to indicate whether the presence of bacteria in the sample is a consequence of an infection, contamination from the environment, or translocation of the bacteria outside the place of its natural existence, which can happen particularly often in patients with an intravenous catheter. Distinguishing between the true bacteraemia and blood sample contamination is a daily challenge in clinical practice, as up to 50% of positive blood cultures are a result of microorganisms transferred from a patient’s skin, as well as the immediate environment of a patient’s or from healthcare workers’ hands [12,26]. The case reports with *S. pettenkoferi* as a potential causative agent of BSIs have been described by several researchers [11,14,15,22,26,29,30]. Vecchia et al. described a case of an 88-year-old woman, admitted to the emergency department (ED) with suspicion of sepsis (the qSOFA; her quick Sepsis Related Organ Failure Assessment score was 2). On the second day of hospitalization, *S. pettenkoferi* was identified in 3 out of 4 blood cultures, collected from different venipuncture sites. The *S. pettenkoferi* strain was resistant to oxacillin (MRCNS; methicillin-resistant coagulase-negative staphylococcal strain), clindamycin, penicillin G, ampicillin, erythromycin, gentamicin, moxifloxacin, and fusidic acid [15]. In the case reports by Hashi et al., a 75-year-old woman was admitted to the ED due to a fall. At the time of the admission, she was afebrile, and a peripheral i.v. catheter was placed for fluids administration. She became febrile twelve hours later, and on the skin where the catheter was inserted, erythema and mild tenderness occurred. *S. pettenkoferi* grew in two blood cultures drawn from separate venipuncture sites. The organisms were susceptible to clindamycin, erythromycin, oxacillin, vancomycin, and trimethoprim/sulfamethaoxazole. The authors assessed that this was most likely a result of CR-BSI [11]. Mammina et al. described the fatal case of bloodstream infection in a 49-year-old male patient admitted to the intensive care unit (ICU) for the surgical treatment of a posttraumatic hydrocephalus. The patient was in coma, with enteral feeding through a percutaneous endoscopic gastrostomy and breathing through a tracheostomy. He underwent the placement of a ventriculo-peritoneal device (VPD), which 10 days later was replaced by an external ventricular drainage, due to the occlusion of the internal device. Due to the suspicion of a bloodstream infection, blood samples were taken. Using the 16S rRNA gene sequencing within 48 h, *S. pettenkoferi* was identified in two blood cultures. Both strains were MRCNS and resistant to erythromycin, clindamycin, fosfomycin, gentamicin, and tobramycin. According to the authors, the presented case report provides evidence of the pathogenic role of *S. pettenkoferi*. The signs of the patient’s deterioration were not related to any other explanation. Indeed, the strains isolated from two blood cultures were molecularly identical [30]. In the study conducted by Park et al., six strains of *S. pettenkoferi* were isolated from blood cultures. After detailed analysis of the clinical features and results of microbiological tests, the authors found that, in only one case, *S. pettenkoferi* was the causative agent of infection (the 72-year-old man with aspiration pneumonia and septic shock, comorbidities, diabetes, and history of alcoholism). In this case, *S. pettenkoferi* was identified in 2 out of 3 peripheral blood cultures and susceptible to oxacillin, penicillin G, ciprofloxacin, gentamicin, vancomycin, teicoplanin, linezolid, clindamycin, erythromycin, and trimethoprim/sulfamethoxazole [14]. Strong et al. described a case of *S. pettenkoferi* causing true bacteraemia in a 73-year-old male patient who was hospitalized in an intensive care unit (ICU). The patient was diagnosed with rapidly progressive sensorimotor neuropathy. As a result of prolonged hospitalization, complications consistent with ventilator-associated pneumonia occurred. The patient developed a sacral pressure ulcer, which progressed to stage 3. A peripherally inserted central catheter was placed. Sepsis was suspected (symptoms occurred: hypotension, hypoxia, and leucocytosis), so two blood samples were collected peripherally forty minutes apart. In two blood culture (after 21 and 72 h from the collection of blood samples) the growth of *S. pettenkoferi* was demonstrated. Both isolates showed susceptibility to vancomycin, clindamycin, rifampicin, and daptomycin, but resistance to oxacillin [26]. In the retrospective study, conducted by Markowska and co-workers, two cases of patients admitted to the emergency department with pneumonia were described. For a 97-years-old woman with chronic renal failure and dialyzed, one blood sample was taken for microbiological testing. The growth of *S. pettenkoferi* was obtained on the first day after sample collection. The strain was resistant to oxacillin, cefoxitin (MRCNS), clindamycin, ciprofloxacin, and levofloxacin; additionally, it was sensitive to trimethoprim/sulfamethoxazole, vancomycin, and teicoplanin. A single blood sample collection makes it difficult, and almost impossible, to correctly interpret the test result. The quick detection of bacterial growth (because of the large inoculum) and lack of alternative causes of infection allowed us to recognize the pathogenic role of *S. pettenkoferi*. The second description regards a 77-year-old man with chronic obstructive pulmonary disease (COPD) and chronic renal failure on chronic haemodyalisis. *S. pettenkoferi* was identified in two culture bottles. The patient was treated empirically with ceftriaxone. Blood cultures, obtained 48 and 72 h later, resulted negative. The strain was sensitive to tigecycline, tetracycline, gentamicin, erythromycin, clindamycin, trimethoprim/sulfameoxazole, ciprofloxacin, vancomycin, teicoplanin, and levofloxacin [22]. Morfin-Otero et al. described a fatal case of a 45-year-old man (intubated, HIV-infected) with a central vascular catheter (CVC). *S. pettenkoferi* was isolated from both of the two blood cultures taken. No improvement in the patient’s condition was observed after administration of the following antibiotics: clindamycin, trimethoprim/sulfameoxazole, azithromycin, and amphotericin [29]. Conversely, in the studies by Argemi et al. and Park et al., *S. pettenkoferi* isolates obtained from blood cultures were defined as not microbiologically relevant [14,31]. Similarly, Mansson et al., in the summary of his observations, explained that *S. pettenkoferi* may be regarded as a probable pathogen in the minority of patients with positive cultures [32]. Ugaban et al. characterized 12 cases of patients (age 38–88; mean ± SD; 60.8 ± 12.8), in which *S. pettenkoferi* was detected in blood samples. *S. pettenkoferi* was cultured from either a single set out of two or more collected from a patient and, in one case, from the only set collected. The time from blood sample collection to detection of the growth of bacteria (time to positivity, TTP) ranges from 17.6 to 43.9 h (mean ± SD; 33.4 ± 12.6) [17]. Hitzenbichler et al. indicated that TTP is an important tool for discriminating between contamination and infection. According to different sources, TTP > 16 h or TTP > 36 h has been associated with CoNS contamination [33]. The possibility of contamination from patient’s saprophytic flora or the environment was also described by Song et al. [13] and Eke et al. [9]. Song and co-workers described a case of 76-year-old male patient who was admitted to hospital for the treatment of recurring pulmonary tuberculosis and had a central venous catheter indwelled. During tuberculosis management and subsequent Stevens-Johnson syndrome occurrence, he developed a sustained and unexplained fever. Two samples of the blood from different lumens of the central venous catheter were taken. The growth of CoNS bacteria was disclosed in two culture bottles. *S. pettenkoferi* was identified in these samples by 16S rRNA gene sequencing. Both organisms showed the same biochemical and genetic features, so they were thought to be the same strain. Authors proved that these isolates have more than 99% similarity with the samples taken from the indoor dust [13]. *S. pettenkoferi* was isolated from two positive cultures. The samples were obtained by catheter. In such cases, results of microbiological tests can be falsely positive (low positive predictive value) because of the possible incident of catheter colonization [34]. Very important information about *S. pettenkoferi* is provided by Eke et al. that analysed the medical records of 80 patients from whom *S. pettenkoferi* was isolated. In 81% of positive blood cultures, *S. pettenkoferi* co-occurred with other bacteria (mixed culture, suggesting colonization) [9]. Signs and symptoms suggestive for sepsis syndrome or septic shock was demonstrated in 78% of patients who had a pure culture. In all these patients, an alternative aetiology has been demonstrated [9,27]. Finally, none of the patients in this study meet the Centers for Disease Control’s criteria for bloodstream infection [9].

## 4. Detection of a True Bacteraemia: Standards, Guidelines, and Best Practices

Several factors should be considered in the proper interpretation of microbiological test results, in order to accept or reject the causative role of *S. pettenkoferi* in one infection [1,9,35]. The misinterpretation of CoNS contaminants, as indicative of true BSI, has implications for both patient care and adequate hospital quality assurance, including antibiotic policy, according to the principle “do not start antimicrobial therapy unless there is clear evidence of infection” [2,36]. Most CoNS positive blood cultures are false positives, as a result of blood sample contamination [1,9,13,27,35,37]. Correct interpretation of the test result requires considering the possibility of:

1. Pre-laboratory errors (disinfection quality of the injection site, amount of blood samples taken, and time of blood samples’ collection);
2. interpretation by laboratory staff (pure or mixed culture and time of a positive blood culture result);
3. clinical criteria (symptoms and predispositions) [27,35,38,39].

One of the possible reasons for blood sample contamination is insufficient skin asepsis [5,35]. More than 20% of the skin microflora may not be damaged/removed during the application of antiseptics on the injection site (location in pilosebaceous and at other places where lipids and superficial cornified epithelium protect them) [35]. Another condition that influence a proper interpretation of the laboratory findings is the amount of blood samples: 2–4 blood sets (one set consists of two bottles with medium enabling growth of aerobic and anaerobic bacteria) and adequate volume of blood sample. The recommended volume of blood to be obtained per culture is 20 to 30 mL (each bottle should be inoculated with approximately 10 mL of blood) [5,27,35,38]. The sensitivity of a single blood culture set is significantly limited. Considering the number of positive blood cultures, a mathematical model has been developed that allows positive predictive value (PPV) estimation, so that, for samples obtained by venipuncture, the PPV was 55% for one positive blood set from one performed, 20% for one positive out of two performed, and 5% for one positive out of three performed. Regarding time for growth (time to positivity), it is considered that the number of positive blood sets within a 24-h culture period has the highest proof of value in interpreting the clinical significance of isolated microorganism. As a rule, bacterial inoculum in a true bacteraemia is higher and grows faster than in contaminated blood culture. When CoNS is isolated, the time from the start of blood culture to the detection of positivity more than 24 h is usually considered a contamination [2,5,27,35]. As Van Aken et al. suggests, the transition of the bacteria to the new environment (blood stream) favours pathogenic strains over commensals [2]. In determining the true bacteraemia, the comparison of isolates of the same species can be very useful. In contaminations, strains of bacteria are frequently genetically unrelated. It is controversial to compare the strains, in terms of virulence markers, but comparison of antibiotics’ sensitivity of CoNS isolated from different blood cultures has been highly predictive. Molecular testing is not practical for standard procedure, as it is cost consuming and lacks proven usefulness [2,35]. In diagnosis of true bacteraemia, the clinical criteria are important; including: (i) the presence of symptoms suggesting blood infection (i.e., fever, chills, and/or hypotension), (ii) the presence of the source of bloodstream infection (secondary bacteraemia), and (iii) characteristics of the patient (immunologically normal hosts, patients with physiological condition impairing defenses, newborns, elder patients, and patients affected by pathological or pharmacological conditions predisposing to infections) [28,40]. Indwelling devices, prosthetic devices, or catheters can be considered as a source of BSI, but merely when the patient has no other defined sources of infection [1,2,11,27,35,40]. It was reported that the true rate of CoNS bacteraemia ranges from 5 to 39.6% [37]. In the study conducted by Cui et al., the 30-day mortality of patients with a true CoNS bacteraemia was up to 12.7%. Thus, the results in a group of patients with chronic renal failure and chronic liver failure was 5.9 and 4.0 times higher than that of patients without these disorders after the onset of CoNS bacteraemia [37]. *S. pettenkofferi*, similarly to other isolated from bloodstream CoNS, may be resistant to antibiotics. Resistance to penicillin, oxacillin, and erythromycin have been reported most common [4,9,14,15,30,41].

## 5. Conclusions

Based on published studies and opinions, it can be stated that *S. pettenkoferi* is a human and animal commensal bacterium. Published reports confirm that *S. pettenkoferi* may represent an opportunistic pathogen and distinguishing between a true bacteraemia and blood culture contamination is not so straightforward [42]. In order to minimize contamination of blood culture rates and improve the accuracy of a bacteraemia diagnosis, the implementing appropriate blood collection procedures, proper assessment, and awareness are extremely critical [35]. The detection of contamination in clinical practice prevents an unnecessary prescription of antimicrobial agents, leading to selection of antimicrobial-resistant organisms, longer hospitalization, and increased costs of medical care. On the other hand, recognizing the true bacteraemia is critical to taking further steps in patient care.
